# Comparison of transverse wires and half pins in Taylor Spatial Frame: A biomechanical study

**DOI:** 10.1186/1749-799X-5-23

**Published:** 2010-03-27

**Authors:** Ashish Khurana, Carlton Byrne, Sam Evans, Hiro Tanaka, Kartik Haraharan

**Affiliations:** 1 Department of Trauma & Orthopaedics, University Hospital of Wales, Heath Park, Cardiff, UK; 2 School of Engineering, Cardiff University, Cardiff, UK; 3 Department of Trauma & Orthopaedics, Royal Gwent Hospital, Newport, UK

## Abstract

**Background:**

The aim of this study was to compare the stiffness characteristics of Taylor Spatial Frame (TSF) fixed with transverse wires and half pins.

**Design & Methods:**

Experiments were carried out at the biomechanics laboratory at Cardiff University. All mechanical testing was performed with a servo hydraulic test frame (MTS 858 Mini Bionix II(R), MTS Corp., Mineapolis, USA). Custom built mounts were used to attach the bone rigidly to the one end of machine and the TSF ring to the other. Rings were fixed with 1.8 mm transverse wires or hydroxy-apatite coated 6.5 mm half pins in 45degrees, 60degrees, 75degrees and 90degrees divergence angles. Bone was loaded with axial load to 400 N and torque to 20 Nm in an indestructible manner. Load/displacement curve data were analyzed for slope and axial and angular displacements.

**Results:**

For larger diameter rings (180 mm), for axial stiffness there was no statistically significant difference between the transverse wires (4 wires with 2 rings) and the half pins (2 pins with 1 ring) (p > 0.05). For 155 mm internal diameter rings, half pins provided statistically higher axial stiffness than transverse wires (p = 0.036). The half pins show significantly more torsion stiffness in both ring diameters (p < 0.05) in comparison to transverse wires. As in axial stiffness, small diameter rings show increased stiffness in torsion. There is increase in axial and torsion stiffness with the increase in the divergence angle between the wires or pins (p < 0.05).

**Conclusion & Clinical Relevance:**

Half pins provide greater stiffness to TSF frames and allow for axial micro motion as well. This work provides a rationale for clinical decision making about the use of tensioned transverse wires in comparison to half pins in construction of a TSF frame

## Background

The Taylor Spatial Frame (TSF; Smith & Nephew, Memphis, Tennessee) is an advanced orthopaedic modality based on a Stewart platform [1]. It is used to treat fractures and correct deformities via an external hexapod fixator that combines ease of application plus computer accuracy[1].

Although TSF is a form of ring fixator, the principles of deformity correction and the material characteristics of the construction are entirely different to the other ring fixators available to Orthopaedic community. Traditionally the ring fixators have been constructed using transverse wires. Transverse wires can cause damage to nerves and blood vessels. Impalement of muscles is often a complication from this technique[2]. In comparison, half pins are safer and easier to apply. The ring fixators work on the principle of allowing axial micromotion with weight bearing which is considered to encourage bone healing[3,4]. The construct of the frame should be sufficiently stiff so as to hold the fracture reduction. On the other hand it requires a fine balance to allow some axial micromotion between the fracture ends to enhance fracture healing.

There is little data on comparative biomechanics of half pins and transverse wires as fixation elements in the TSF. There are some studies in literature evaluating the clinical outcome of TSF[5], but there are none evaluating the biomechanics of TSF.

The aim of this study was to compare the axial and torsion biomechanics of TSF rings fixed with half pins and transverse wires. This study was designed to address the following research questions:

1)Comparison of axial and torsional stiffness characteristics of TSF with half pins and transverse wires.

2)Variation of the stiffness characteristics with variation in the divergence angle from 45 to 90 degrees for both wires and half pins

3)Variation of the stiffness characteristics with change of ring size from 155 mm to 180 mm internal diameter.

## Materials and methods

The experiments were carried out at the Biomechanics laboratory, School of Engineering, Cardiff University. All mechanical testing was performed with a servo hydraulic test frame (MTS 858 Mini Bionix II^®^, MTS Corp., Minneapolis, USA).

**Figure 1:**
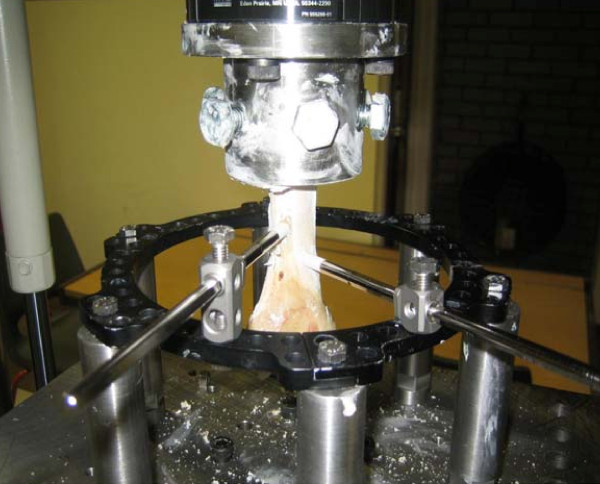
**A typical test setup suing half pins**. The bone is held by the mount at the top end of the test frame and the ring is held rigidly by the bottom end of the frame. The weakest link in the setup is wires or pins connecting the bone to the rings. When load is applied to the bone from the top end it will displace depending on how rigidly it is held by wires or pins.

Custom built mounts were used to attach bone rigidly to the top end of the machine. Cadaver calf tibiae were used for the experiments. Bone was fixed into the mount using centralising bolts (figure 1). To avoid point application of force and to prevent any toggle on force application, the free space around the bone in the mount was filled with a polymer filler. This provided an absolute rigid fixation of bone to the mount.

**Figure 2:**
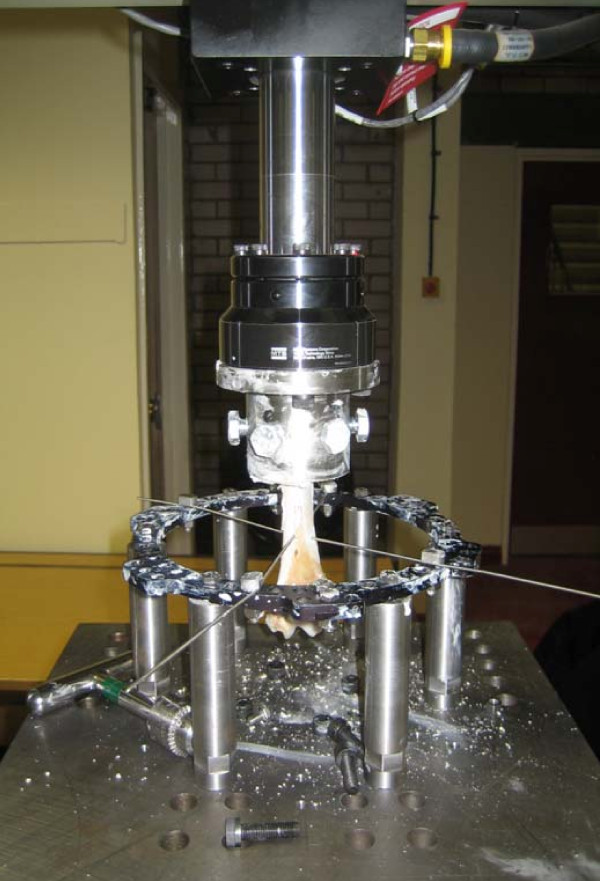
**A typical test setup using transverse wires**.

TSF ring was attached using another custom built mount to the other end of the testing machine. The mount used to hold the rings had modularity to enable attachment of different size rings. Rings were fixed with 1.8 mm transverse wires or hydroxy-apatite coated 6.5 mm half pins. Half pins were inserted after predrilling. Tests were performed with the wires or pins in 45°, 60°, 75° and 90° intersection angles. All tests were also repeated with 2 ring sizes: 155 and 180 mm internal diameter. Figure 2 shows a typical test set up with the MTS test frame using transverse wires.

Transverse wires used were 1.8 mm stainless steel wires, tensioned to 110 kg. A standard calibrated dynamometric tensioning device (Smith & Nephew, Memphis, Tennessee) was used for tensioning the transverse wires. Tests were also performed with an additional ring mounted with 2 transverse wires, as is common in clinical practice. These 2 rings, mounted with 2 wires each, were connected to each other with rods and acted as a single assembly. For tests using half pins, 6.5 mm (section diameter) hydroxy-apatite coated half-pins were fixed onto the rings using rancho cubes. Half pins were inserted bi-cortically. The wires or pins crossed in the centre of tibia and the tibia was positioned in the centre of the ring. New wires were used for all experiments. Half pins were replaced only if there was any visible deformation in them.

Component variables evaluated were ring diameter (155 mm and 180 mm) and wire or pin divergence angle (45 degrees, 60 degrees, 75 degrees and 90 degrees). This resulted in 24 constructs based on 2 ring sizes, 4 divergence angles and 3 configurations (half pins, 1 ring fixed with 2 transverse wires and 2 rings fixed with 2 transverse wires each). Each construct was evaluated for axial and torsional biomechanics. The tests were repeated thrice on each fixation and the results were averaged. Experiments were performed in a random sequence.

Based on the literature review [5-9] and to apply loads in the clinical range, the bone was loaded with axial load to 400 N and torque to 20 Nm in an indestructible manner. Axial load was applied over 60 seconds and 5 mm displacement was set as the maximum permissible displacement. Similarly, torque was applied over a period of 60 seconds with 30 degree angular displacement as the maximum limit. Torsional stiffness was tested without any coupled axial preload.

Main Outcome measures: axial and angular deformation characteristics as a result of axial and rotational (torque) load respectively were compared for the described constructs. Displacement of the bone in relation to the pre - load position was recorded by the MTS test frame. Load/displacement data was obtained for each individual ring fixation and the curves were analyzed for slope, axial and angular displacements. The slope of the regression line of these average data points is defined as the stiffness (load/deformation). Stiffness values for various fixations were compared. Data was stored using excel and was analysed on SPSS software (Version 14, SPSS Inc. Chicago, Il). The data were analysed using an analysis of variance (ANOVA) and individual differences were determined using a post hoc test. Students t test was used to compare the corresponding stiffness values of rings fixed with half pins and transverse wires between two specific groups. A p value of ≤ 0.05 was considered to be significant.

## Results

### Axial Stiffness

Stiffness was calculated from load displacement curve by linear regression between 250 and 300 N loading. This provided an intermittent linear portion in the curve[6] which corresponds to the clinical range of loads applied to the lower limb bones on weight bearing. As described earlier, tests were carried out on ring constructs made using 2 half pins in comparison to those constructed using transverse wires. Use of one ring in transverse wire construct was also compared with a 2 ring constructs using transverse wires (with an accessory ring). Structural failure was not observed in any specimen. A non linear load displacement behaviour was observed for all specimens in the test range.

Table 1 shows the comparative axial stiffness of TSF rings fixed with transverse wires and half pins in varying divergent angles. The increase in stiffness between a transverse wire (with accessory ring) construct and a half pin construct ranged from 20.4% to 50%. The stiffness of the fixation increased with increasing intersection angle between the wires or pins. This was true for both wires and the pins. Constructs with divergent angle of 90 degree were found to be most stiff in both the ring diameters (p < 0.05) (table 1).

**Table 1:** Axial stiffness of rings

	180 mm rings			155 mm rings		
**Divergence Angle**	**Half pins (1 ring)**	**Transverse Wires (2 rings)**	**Transverse Wires (1 ring)**	**Half pins (1 ring)**	**Transverse Wires (2 rings)**	**Transverse Wires (1 ring)**

90°	98.04 (±2.03)	102.04 (±1.28)	68.49 (±0.39)	200 (±2.61)	138.88 (±2.08)	80.64 (±0.33)

75°	89.28 (±2.37)	90.59 (±1.85)	67.56 (±0.83)	192.3 (±1.27)	128.2 (±1.15)	78.12 (±1.93)

60°	74.62 (±1.46)	79.36 (±2.76)	52.08 (±2.18)	151.51 (±0.19)	125 (±0.08)	79.36 (±2.81)

45°	63.29 (±0.37)	65.44 (±2.31)	51.02 (±1.49)	116.27 (±1.68)	92.59 (±1.27)	83.33 (±2.89)

All values in N/mm and brackets show SD. For larger diameter rings (180 mm) there was no statistically significant difference between the transverse wires (with accessory ring) and the half pins (p > 0.05). Both, half pin and transverse wires constructs with accessory rings were significantly stiffer than a single ring fixation using 2 transverse wires only (p = 0.017). For 155 mm internal diameter rings, half pins provided statistically higher axial stiffness than transverse wires with accessory rings (p = 0.036).

**Figure 3:**
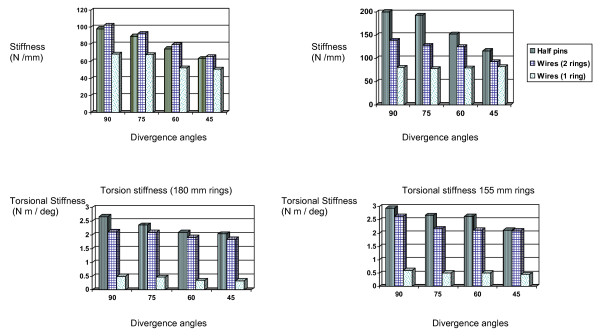
**a: Axial stiffness of 180 mm rings**. b: Axial stiffness of 155 mm rings. c: Torsion stiffness (180 mm rings). d: Torsion stiffness 155 mm rings.

Force required to produce 1 mm displacement was also analysed for all the configurations. This reflects clinically important range of displacements and the associated fixator stiffness[6]. Results are as per table 2. Figures 3a and 3b compare the axial stiffness in varying divergence angles, as shown in Table 1.

**Table 2:** Force for 1 mm displacement

	180 mm rings		155 mm rings	
**Divergence Angle**	**Half pins (1 ring)**	**Transverse Wires (2 rings)**	**Half pins (1 ring)**	**Transverse Wires (2 rings)**

90°	188.1 (±3.27)	198.8 (±1.46)	209 (±0.35)	195.5 (±1.63)

75°	169.19 (±1.77)	173.46 (±2.49)	204 (±3.18)	184.3 (±3.51)

60°	149.77 (±1.39)	155.66 (±2.83)	178.8 (±2.67)	178.8 (±1.35)

45°	129.35 (±0.78)	124.37 (±3.71)	164.4 (±1.93)	150.1 (±2.47)

All values in N and brackets show SD. There was no statistically significant difference between the half pin and transverse wires (with accessory ring) assembly (p > 0.05). However, there was a significant increase in force required to allow 1 mm movement in the smaller (155 mm) ring size (p = 0.038).

### Torsional Stiffness

Torsional stiffness (table 3) was calculated as regression from the torsional moment and angulation data between 4 and 7 Nm torque. This was calculated as slope of the graph obtained between torsion load and angular displacement.

**Table 3:** Torsion stiffness

	180 mm rings			155 mm rings		
**Divergence Angle:**	**Half pins (1 ring)**	**Transverse Wires (2 rings)**	**Transverse Wires (1 ring)**	**Half pins (1 ring)**	**Transverse Wires (2 rings)**	**Transverse Wires (1 ring)**

90°	2.67 (±0.12)	2.11 (±0.05)	0.49 (±0.04)	2.94 (±0.07)	2.63 (±0.08)	0.60 (±0.04)

75°	2.36 (±0.07)	2.09 (±0.09)	0.46 (±0.06)	2.67 (±0.10)	2.17 (±0.11)	0.51 (±0.03)

60°	2.09 (±0.08)	1.91 (±0.11)	0.35 (±0.02)	2.63 (±0.04)	2.11 (±0.04)	0.51 (±0.07)

45°	2.04 (±0.10)	1.84 (±0.08)	0.34 (±0.05)	2.11 (±0.05)	2.09 (±0.07)	0.45 (±0.06)

All values in Nm/deg and brackets show SD. The half pins show significantly more torsion stiffness in both ring diameters (p < 0.05) in comparison to transverse wires. There is increase in stiffness with the increase in the divergence angle as well (p = 0.048). As in axial stiffness, small diameter rings show increased stiffness in torsion. Single ring fixation with transverse wires (without any accessory rings) provides significantly weak construct in both ring diameters and all divergence angles (p = 0.008). For 180 mm rings, the torsion stiffness of half pins construct is between 9.4% to 26.5% more than the transverse wires (with accessory ring) construct. Similarly, for 155 mm diameter rings, there was an increase between 9.5% and 24.6% for half pins in comparison to transverse wires (with accessory ring) construct.

## Discussion

**Figure 4:**
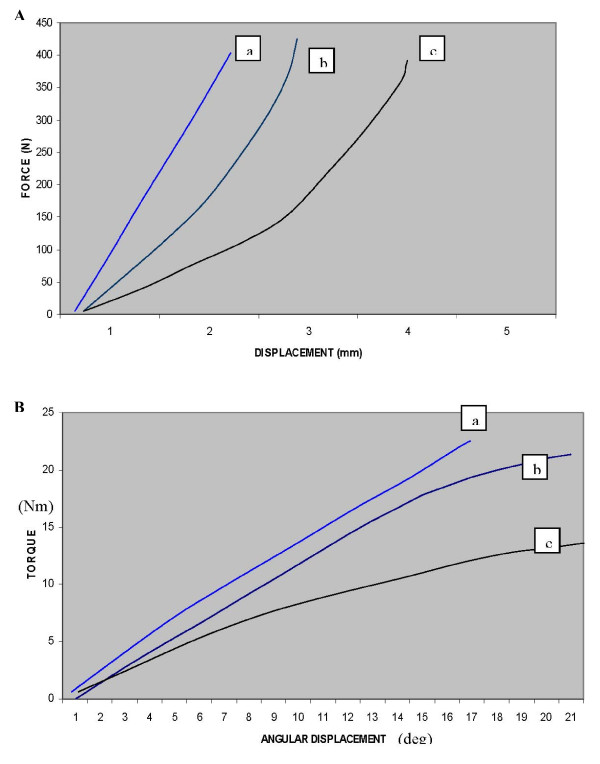
**a-b: Load displacement curves for the three configurations with axial and torsion loading (with divergence angle 90 degree and 155 mm TSF ring)**.

This study suggests that the transverse wires (with accessory ring) and half pins provide comparable axial stiffness for 180 mm rings. Based on Table 1, for all respective divergence angles, there is no statistically significant difference between the stiffness of half pins and transverse wires (with accessory rings) for 180 mm rings. As per Table 1, the axial stiffness of a ring fixed with half pins in 90 degrees is 98.04 ± 2.0 N/mm and that with wires was 102.04 ± 1.3 N/mm. In clinical practice, because of anatomical constraints it is not possible to fix a ring with wires crossing at 90 degrees. But it is possible to put pins in 90 degrees. The maximum angle of wires divergence is 60 degrees in clinical practice [2,5,10,11]. Stiffness achieved with this construct was 79.36 ± 2.8 N/mm, when using an accessory ring. There was a significant difference when this is compared to half pins in 90 degrees (i.e. 98.04 ± 2.0 N/mm) (p = 0.028). Hence, the versatility and modularity of half pin system enables to achieve stiffness more than that possible with a transverse wires construct. The axial stiffness provided by transverse wires using a single ring is statistically much inferior to either of the other constructs. Figures 4a-b show the typical load displacement curves for the three configurations with divergence angle of 90 degree.

Though the pins provide comparable or more stiffness than transverse wires, they allow some axial movements as well. As shown in Table 2, 209 ± 0.4 N and 188.1 ± 3.3 N load was required to produce 1 mm axial movement with half pins fixation at 90 degree in 155 and 180 mm rings respectively. This load is in clinical range and is applied on weight bearing. Hence, pin fixation can also provide axial micromotion similar to that seen in transverse wires.

The half pins show significantly more torsion stiffness in both ring diameters (p < 0.05) in comparison to transverse wires as shown in Table 3 and figures 3c and 3d. There is an increase in stiffness with the increase in the divergence angle as well, which was statistically significant (p < 0.05).

Table 1 and 2 show a progressive increase in axial stiffness with increase in divergence angles for both sized rings. This is true for both half pins as well as the transverse wires. Similar increase in torsional stiffness is also seen as demonstrated in Table 3. Podolsky and Chao[12] concluded the same for axial stiffness in their experiments. However they also concluded that fixators with wires crossing at 45 had significantly greater stiffness in torsion compared to those with 90 deg crossing wires. In the experiments by Roberts et al, a considerable reduction in the axial and torsion stiffness with decreasing wire divergence angle was observed[5]. Our findings match that of both these authors with regard to axial stiffness. However, in our tests the torsional stiffness also increased with increasing divergent angles, for both transverse wires and the half pins. These are in conformity with Roberts et al[5].

When the ring size is decreased to 155 mm the axial stiffness of the half pin construct is significantly more than the transverse wires construct (with an accessory ring). With smaller ring size, half pins are significantly stiffer than transverse wires (with accessory ring), whereas with larger diameter the two were comparable as demonstrated in Table 1, 2, 3. Further evaluation of effects of ring size over a broad spectrum may be warranted before any definite conclusions could be drawn. But the current results lead to a hypothesis that pins may loose relative stiffness benefits in larger ring sizes and hence should be avoided for obese subjects, which require larger ring sizes. This is due to increase in the bending moment of the half pins, which act as cantilever beams, in larger rings. This may increase the stresses at the pin bone interface, leading to pin loosening and decreased stiffness. Ring diameter has been shown to have significant effect on stiffness[6,13-15]. Unfortunately the ring diameter is dictated by the size of the patient and the smallest diameter should be used giving adequate clearance to soft tissues[16]. Decrease of the construct stiffness with increase in ring diameter is attributable to the fact that deflection of a wire subjected to a specific load is dependant in part on functional length of the wire[6,12,14].

Wire tension is a major influence on the stiffness of ring fixators[17]. In this study new wires were used for each test. In clinical practice, there is more probability of wires going loose, leading to loss of stiffness[17]. Half pins do not need tensioning and with decreasing incidence of infection with surgical care and hydroxy-apatite coating[18,19] stiffness of a pin based fixator would be expected to be maintained during clinical use.

There are some studies in literature evaluating the clinical outcome of TSF[20-22], but there are none evaluating the biomechanics of TSF. Amongst biomechanical studies on other ring fixators, all but one study found in English literature are based on frames[5]. This does not eliminate the confounding effects of frame assembly, connecting struts or rods, ring spacing and the interface characteristics of fixation elements used for multiple rings[5]. The design in our study was established so as to evaluate only wire and pin behaviour as fixation elements in TSF with elimination of other confounding variables. In our experiments, bone was rigidly attached to one end and the ring was attached to the bottom end of the MTS frame. The set up ensured that the only weak link in the assembly was the wire or half pin connection between the ring and the bone.

It is very difficult to compare the results of different studies on ring fixators because not only the fixator construction characteristics but also the loading modes are usually different[8]. The difference in the results of various researchers in the biomechanics of ring fixators can be attributed to difference in the modality of their tests.

It can be difficult to appropriately determine construct stiffness for non linear behaviour. The data can be transformed logrythmatically to determine stiffness at multiple points along the load displacement curve with an assumption of linearity at each point[12,13]. The stiffness can also be determined at intermittent or terminal linear portions of the graph[6]. Cross et al observed that this may bias the data towards higher stiffness values but provides a valid means of comparison between constructs[6]. In this study, stiffness was calculated from load displacement curve by linear regression between 250 and 300 N loading. Windhagen et al used a similar methodology but a different range (300 to 350 N) [9]. 250 to 300 N load was used for analysis in this study as we believed that this corresponds closely to the loads applied on the lower limbs on weight bearing.

### Limitations and further research

This study had some potential limitations. All tests were performed on calf bones. There could be a difference between the biomechanical characteristics of human and calf bones. Orienti has concluded from his work that the in vitro model does not realistically simulate the behaviour of external fixation pins implanted in ex-vivo bone[23]. The cadaveric bones may also have anthropometric variations. An attempt to decrease this effect was made by obtaining the bones from same aged cadaveric calf from a single population. All experiments were repeated thrice and were performed in a random order to compensate for any variation.

New transverse wires were used for all tests. However the same half pins were repeatedly used to limit the costs. They were discarded only if any damage was obvious. This may affect the results to some extent. Random order of tests and repetition of tests decreased the bias in the results.

The stiffness was tested under experimental conditions and the bone was centrally fixed within the ring. In clinical practice this may often not be possible due the bone shape and the surrounding soft tissues. Podolsky and Chao illustrated that eccentric placement of bone within the rings has no adverse effects[12]. The authors also believe that with rigid fixation of the bone with the MTS frame, the constructs were constrained so that the bone could not move sideways under axial loading. This affected the half pin constructs more than the wire constructs since they are not symmetrical, creating a bias in favour of the half pins.

Only axial and torsional stiffness was tested in our study. These two are the most crucial forces acting on the lower limb fixators. Bending stiffness is more dependant on the connecting rods/struts between the rings rather than the fixation elements like transverse wires and half pins. Moreover, tests for evaluating bending stiffness were not possible on the available MTS 858 Mini Bionix machine.

Torsional stiffness was tested without any coupled axial preload. Further work can be performed to analyse the torsional stiffness characteristics with applied axial preloads to simulate weight bearing along with torsional stress.

These experiments were performed in a controlled manner and provide an estimate of stiffness. Bone loading during mobilisation and physiological conditions can be eccentric and combination of various forces. The study has not evaluated the biomechanics of the combined use of half pins and transverses wires in a hybrid frame. This work provides a rationale for clinical decision making about the use of tensioned transverse wires in comparison to half pins in construction of a TSF frame.

## Competing interests

The authors declare that they have no competing interests.

## Authors' contributions

AK coordinated the study, carried out the experiments, analysed the result and drafted the manuscript. CB and SE designed the setup and helped to perform the tests. HT & KH conceptualised the study, analysed the results and helped to draft the manuscript. All authors read and approved the final manuscript.
